# Perceptions of complementary/alternative medicine use and influence on evidence-based asthma medicine adherence in Malaysian children

**DOI:** 10.1038/s41533-019-0118-x

**Published:** 2019-02-25

**Authors:** Siti Nurkamilla Ramdzan, Hilary Pinnock, Su May Liew, Nursyuhada Sukri, Hani Salim, Nik Sherina Hanafi, Norita Hussein, Julia Suhaimi, Ping Yein Lee, Ai Theng Cheong, Azainorsuzila Mohd Ahad, Steve Cunningham, Ee Ming Khoo

**Affiliations:** 1 Department of Primary Care Medicine, Faculty of Medicine, University of Malaya, Kuala Lumpur, Malaysia; 2 NIHR Global Health Research Unit on Respiratory Health (RESPIRE), Usher Institute of Population Health Sciences and Informatics, University of Edinburgh, Edinburgh, UK; 3 Universiti Putra Malaysia, Selangor, Malaysia; 4 Ministry of Health, Putrajaya, Malaysia

## Abstract

Complementary and alternative medicine (CAM) is widely used especially in Asia including for childhood asthma. The use of CAM could influence adherence to evidence-based (E-B) medicine. We explored the views of carers of Malaysian children with asthma regarding the use of CAM for childhood asthma, and its relationship with self-reported adherence to E-B medicine. We used a screening questionnaire to identify children diagnosed with asthma from seven suburban primary schools in Malaysia. Informed consent was obtained prior to the interviews. We conducted the interviews using a semi-structured topic guide in participants’ preferred language (Malay, Mandarin, or Tamil). All interviews were audio-recorded, transcribed verbatim and coded using Nvivo. Analysis was performed thematically, informed by the Necessity-Concerns Framework. A total of 46 carers (16 Malays, 21 Indians, 9 Chinese) contributed to 12 focus groups and one individual interview. We categorised participants’ as ‘Non-CAM’; ‘CAM’; or ‘combination’ user. Cultural practices and beliefs in the efficacy of CAM resulted in widespread use of CAM. Most carers used CAM as ‘complementary’ to E-B medicine. Concerns about dependence on or side effects of E-B treatment influenced carers’ decisions to rely on CAM as an ‘alternative’, with an important minority of accounts describing potentially harmful CAM-use. Healthcare professionals should discuss beliefs about the necessity for and concerns about use of both E-B medicine and CAM, and provide balanced information about effectiveness and safety. The aim is to improve adherence to regular E-B preventer medication and prevent delays in seeking medical advice and harmful practices associated with CAM.

## Introduction

Childhood asthma is one of the most common chronic diseases among children.^1^ It poses a significant healthcare burden worldwide and is one of the top ten causes of disability-adjusted life years among children aged 5–14 years.^2^

Evidence-based medicine (E-B medicine) as recommended by global guidelines for asthma management can substantially reduce this burden.^3^ Good control of asthma symptoms and prevention of attacks is achievable for most children with regular inhaled corticosteroids, and bronchodilators for relief of acute symptoms.^3–5^ These treatments, however, only work if they are taken, and adherence to regular E-B medicine is notoriously poor.^6,7^ A widely accepted model that helps understand key reasons for poor adherence is the necessity-concerns framework which holds that patients’/carers’ use common-sense judgement when they decide to use treatment (or not). People balance their perception about the necessity for a treatment with their concerns about possible adverse consequences.^8^

A factor that may influence parental decisions about adherence to E-B medicine is their beliefs and use of complementary and alternative medicine (CAM).^9^ The use of CAM is increasing worldwide but is widely used and well accepted in Asia.^10–12^ A survey done in a primary care clinic in Malaysia revealed almost half of patients with asthma used CAM to complement E-B medicine, and most did not tell their doctor about their use.^12^ Further, Malaysians from Malay, Indian and Chinese cultures may use CAM as a ‘substitute’ rather than as ‘complementary’ to E-B medicine, raising particular concerns about safety.^13^ Regular use of CAM may reduce adherence to E-B preventer medicines, and use of CAM to relieve acute symptoms may cause a delay in seeking treatment during asthma attacks.^14,15^ However, most of the studies have been done in clinical or healthcare settings, selectively overlooking people who seldom or never seek treatment using E-B medicine.^16^ We therefore aimed to address this gap by exploring the views of carers regarding the use of CAM for asthma, and the relationship of CAM use with self-reported adherence to E-B medicine among school children with asthma in Malaysia (a multi-cultural society where CAM use is widespread amongst some communities).

## Results

We approached the carers of 65 children identified as having asthma. A total of 46 carers took part in 12 focus groups and one in-depth interview between 4th April 2016 and 15th November 2016. A repeat interview was conducted for two participants in one focus group discussion (one was not able to complete the first focus group for personal reasons and another whose views warranted further clarification as they were deviant from others in the group) for better understanding about their beliefs and CAM practices.

Table 1 shows the socio-demographic background of the participants. Most of the carers were mothers of the children; only a quarter of the children had good control of asthma according to GINA guidelines.

**Table 1:** Socio-demographic background of participants (*n* = 46)

Demographic characteristics	Malay (*n* = 16)	Chinese (*n* = 9)	Indian (*n* = 21)
Age range (year)	33–51	31–62	30–68
Female	16	9	17
Male	0	0	4
*Education background*			
Primary	1	2	1
Secondary	9	7	9
Tertiary	6	0	6
Missing	0	0	8
*Carer relationship with children*			
Parents	16	8	19
Grandparents	0	1	2
*Use of CAM for asthma*			
Non-CAM user	4	2	8
CAM user	1	2	1
Combination user	11	5	12

Table 2 shows the different types of CAM used by the participants. Some of the CAM choices were culturally specific e.g. Chinese preferred Chinese traditional medication, Indians preferred ayurvedic treatment, and Malay preferred Malay and Muslim CAM such as honey, black cumin seeds and Malay healing rituals. However, we also observed cross-cultural use of CAM between different ethnic groups.

**Table 2:** Types of CAM used by participants for asthma treatment

Types of CAM	Examples
*Malay*	
Supplements	Vitamins, fish oil extract
Traditional or herbal medicine	Medicated ointment (camphor or methol-based), bird’s nest, ayurvedic medication, sea cucumber
Homoeopathy or naturopathy	Honey, warm water, dates, stevia, black cumin seeds (*nigella sativa*), pomegranate juice
Spiritual/religious healing	Healing rituals, meditation
Others	Induce vomiting, air ioniser
*Chinese*	
Supplements	Vitamins, fish oil extract
Traditional or herbal medicine	Bird’s nest, Chinese traditional medication, crocodile meat, medicated ointment
Homoeopathy or naturopathy	Red/black bean soups, bone broth
Relaxation/Spiritual healing	Breathing exercise, breathing through wet towel
*Indian*	
Supplements	Vitamins, fish oil extract
Traditional or herbal medicine	Ayurvedic medication, Noni juice, chinese traditional medicine, crocodile meat, basil leaves medicated ointment
Homoeopathy or naturopathy	Honey, warm water

To understand the use of CAM for asthma and its relationship with self-reported adherence to E-B medicine, the participants were divided into three groups according to the carers’ pattern of CAM use for their children.^17^

1. Non-CAM users: not current users of CAM (though may have used CAM in the past).
2. CAM users: current users of CAM though may use E-B medicine as a last resort.
3. Combination users: current users of both CAM and E-B medicine.

**Fig. 1:**
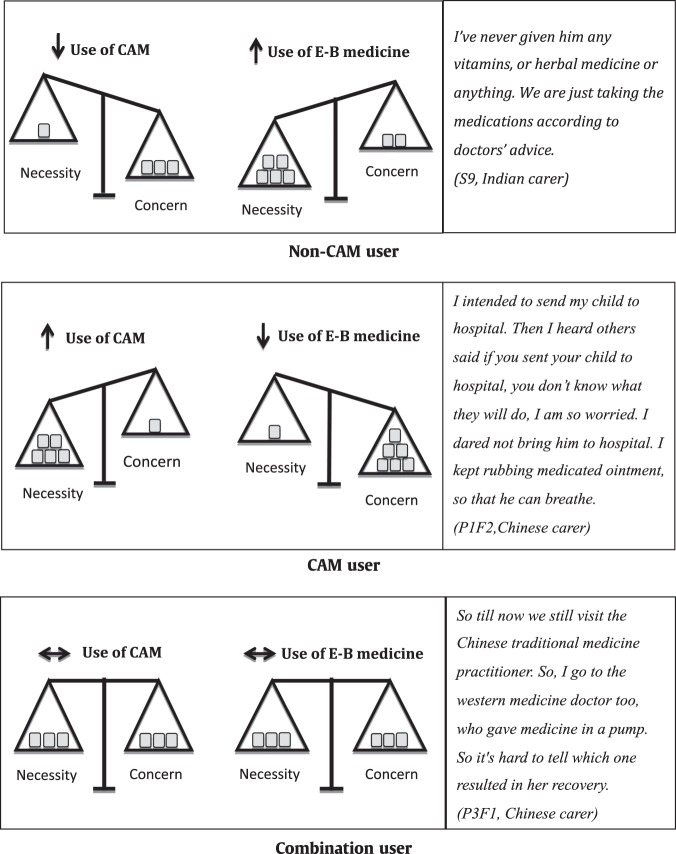
Necessity-Concern framework and the balance between CAM and E-B medicine according to the groups

Figure 1 illustrates the necessity-concern framework as it applies to the use of CAM and E-B medicine according to these groups. Table 3 provides additional quotes to demonstrate the relationship of CAM use with E-B medicine according to these groups.

**Table 3:** Additional quotes from participants regarding the use of CAM according to groups

*Non CAM user*	
Necessity of EB-medicine	When he makes the sound and then straight to the hospital because my late father died from asthma, that’s why sometimes I get phobia. (M1, Malay carer)
Concern of CAM	We bought a protein from Amway once. It was a good medicine but he couldn’t take it… He couldn’t take it. He just vomits out that protein, so we stopped giving that (S9, Indian carer)
*CAM user*	
Necessity of CAM	Since I am worried of him, not sure if he will have an attack, I will give him a wet towel and ask him to tell the teacher if he feels uncomfortable and to wet the towel, cover and breathe in and out five times. (P1F2, Chinese carer)
Concerns of EB-medicine	I do not use the aerochamber (inhaler)... because I am afraid he would be addicted, whenever he gets it he will want the aerochamber, because when he goes to the hospital to get oxygen (nebuliser), he doesn’t… there is no reduction, they will add more with drip. (I, Malay carer)
Necessity of E-B medicine as a last resort	I’ll watch her carefully. If she’s about to vomit, she’ll bang herself on walls and the vomits will be coming out through nose. Once she starts to bang on walls, will bring her straight to hospital. (RP1, Indian carer)
*Combination user*	
Necessity of CAM and EB-medicine among carers who favoured CAM over EB-medicine	I feel that the supplements are good… I gave pomegranate juice, I gave it for a long time.. and he did not get it (asthma symptoms and attacks)… it is not so often anymore, he doesn’t use his inhaler anymore, it has expired… so we are just on standby, afraid he would get it suddenly. (AM1, Malay carer)
Necessity of CAM and EB-medicine among carers who favoured E-B medicine over CAM	He will start to cough keh,keh,keh then I know, if we leave it, it will worsen, give supplement to improve immunity of his body. (Z1, Malay carer) My father-in-law bought that crocodile meat for £15 for my children. Told me to dry them and fry it like salted fish. (K7, Indian carer)

### Non-CAM users

Non-CAM users decided against CAM use because they believed CAM was not necessary and had some concerns with side effects of CAM or intolerability of the child to taking CAM.

My father's friend once suggested us to try crocodile meat, saying that it cures asthma. So, my father bought it from a Chinese shop. and we gave it to our kids to try… after eating that they couldn't sleep at all at night because of the ‘heat’ it caused. It was very ‘heaty’ even with the air conditioner switched on. (PK4, Indian carer)

The belief of non-CAM users regarding the use of E-B medicine was the opposite of their belief about CAM. Non-CAM users believed that their child needed E-B medicines and they expressed fewer concerns about its use. Their self-reported adherence to E-B medicines was good because of past worrying experience of asthma, and/or past good experience with effective E-B medicines use so that they believed it was a necessity.

Salbutamol, yes, that is if he coughs, has a cold, because we want to reduce and dilute his phlegm. If it is the usual cough or cold medications, they definitely will not work. He needs to have the salbutamol to clear his lungs. (S4, Malay)

Some carers did not have any concerns with the use of E-B medicine, though some described difficulty in administering E-B medicine e.g. unpalatable taste.

It is difficult to persuade him to take medication (due to taste). (A9, Indian carer)

### CAM users

CAM users expressed the opposite opinion about CAM which they believed was a necessity with few disadvantages, in contrast to their beliefs about E-B medicine which they had significant concerns and which they believed was not necessary. These perceptions led to substitution of E-B medicine with CAM and self-reported poor adherence to E-B medicine.

CAM users believed that CAM was effective; some believed that it could actually cure asthma.

You have to find something to take together with crocodile meat. That medicine (crocodile meat medicine) is actually quite cheap, while crocodile meat is more expensive. Give him to eat three times continuously and asthma will be cured. (P3F2, Chinese carer)

Carers in this group used CAM as first line treatment to relieve asthma symptoms and asthma attacks. They described the strategies they recommended when their child was having asthma symptoms or an attack at school (for example: induced vomiting and breathing through a wet towel).

A way that he does at school when he cannot breathe, he will go to the toilet and induce vomiting, he will vomit everything, only then it is easy for him to breathe. (I, Malay carer)

CAM users did not use preventer medication for asthma because they believed that E-B medicine caused side effects, or were concerned about engendering dependency. They believed that frequent use of E-B medicine would lead to increase dosage of medication their child required and hamper attempts at tapering off the medication. Other concerns among primarily CAM users regarding E-B medicine were related to their beliefs about ineffectiveness and/or past bad experience with the use of E-B medicine.

It happened when his asthma was very serious, I brought him there and the medication is not suitable for him. You know…. He felt bad, felt faint. (P1F2, Chinese carer)

CAM users chose to use CAM even during asthma attacks and would only use E-B medicine as a last resort.

I will reverse him like a baby, I will hold his feet, his father will hold him at the bottom, I will smack his back, let him breathe like normal first, if that still doesn’t work, if he is too pale, we will reverse him to a standing position and smack his back. If that doesn’t work, I will send him to the hospital. (I, Malay carer)

### Combination user

Carers in this group decided to use both CAM and E-B medicine concurrently. Within this group, however, carers described a range of beliefs about CAM and E-B medicine which determined the choices they made as they balanced their perceived necessity and concerns about the two treatment modalities.

Concerns with E-B medicine appeared to be the main reason that resulted in combination users favouring CAM most of the time. These carers were worried about the side effects of E-B medicine or doubtful about its effectiveness. Because of these concerns, carers used CAM to reduce the use of E-B medicine, impairing adherence to regular therapy. However, they were still using some E-B medicine because they had not found the ‘ideal’ CAM or were worried about stopping using E-B medicine completely.

Because from the time my child was four years old, she used inhalers until a year ago, I feel like her (usage of) pump increased, her dose increased… it made me sad. People say that the more you take medicine, the less you should need it, the dose should decrease, the daily frequency should decreased but she needed to take it more and more often, so that is why I tried the other products that are in the market, until I found stevia at last… that is what worked for my child. (S1, Malay carer)

Combination users who, on balance, favoured E-B medicine over CAM were using CAM to complement E-B medicine. They believed in the effectiveness of E-B medicine and had less concern about its use. However, they believed CAM was also necessary reflecting their cultural beliefs and practices. Some carers had ‘hot and cold’ beliefs—they believed that asthma is ‘cold’, thus their child needed ‘hot’ medication. Some carers believed CAM offered modalities that were not provided by E-B medicine, such as improvement in general health and the immune system that could indirectly reduce asthma symptoms and attacks. Some carers used CAM because of cultural influence such as recommendation by family members and friends.

Double boil ginseng... It seems to be more ‘heaty’… When they feel ‘heaty’, they won’t have asthma… Yes, it (asthma) is ‘cold’. (P2F3, Chinese carer)

Strong concerns about the side effects of E-B medicine or effectiveness of CAM for asthma underpinned the alterations combination users made to their child’s asthma action plan. Some carers preferred to use CAM as first line treatment or use it concurrently with E-B medicine.

I’ll give warm water first. If they cough worsens even after giving warm water, then I’ll give him inhalers. Usually he’ll get better after using the inhalers, he’ll be back to normal. (KO4, Indian carer)

If he starts to cough a lot, I give him honey immediately. He takes 2 teaspoons a day, but I’ll give more if he coughs. But if it is under control that is all that I give. I give that but I also give salbutamol every 8 hour as a precaution. (M2, Malay carer)

## Discussion

Our findings demonstrate how carers balance their beliefs about E-B medicine and CAM to decide on management strategies for their children. Cultural practices and beliefs in the efficacy of CAM resulted in widespread use of CAM in all three of the ethnic communities in Malaysia. Most carers use CAM as ‘complementary’ to their use of E-B medicine. Concerns about dependence on or side effects of E-B treatment influenced decisions to rely on CAM as an ‘alternative’, though most carers would seek E-B medicine in an emergency. (albeit sometimes as a last resort potentially risking serious delays)

Concern about side effects is a recognised barrier to adherence with E-B regular medication in all communities.^14^ We found that attitudes and beliefs about CAM are an important influence on how carers weight their perception of necessity and concerns of the two treatment modalities and decisions about how they manage their child’s asthma. Questionnaire surveys in the US have similarly reported an association between children’s CAM-use and carers’ concerns about E-B medicine in majority,^18^ and minority American communities (African-Americans,^15^ Latino^15^ and Vietnamese^19^). Similar findings have been reported in studies conducted elsewhere.^20–22^

A specific concern was fear of dependency to E-B medicine. This is widely reported in studies of CAM-use in children, from all backgrounds^22–25^ and was quantified as being a concern for four fifths of the respondents in a survey conducted in a Malaysian paediatric outpatient clinic.^24^ Serious side effects of steroids (such as adrenal insufficiency) are uncommon,^3,26^ but carers’ concerns about the side effects of E-B medicine are not totally unfounded, as oral and inhaled corticosteroids have been reported to cause a number of troublesome adverse effects,^27^ though the benefits normally outweigh the risks.^3^ Thus, healthcare professionals should listen to and address patients concerns about side effects to improve their patients’ adherence.

In line with previous studies,^10,19,28,29^ cultural norms influenced the use of CAM among carers in our study. Many of these studies were from minority communities in the US or Europe with African, Asian or Latin American ethnic background.^19,29,30^ It is likely that the culture to use CAM was strongly influenced by their ethnicity as high prevalence of CAM use has been observed among these ethnic groups in their own countries.^11,12^ However, participants from the minority communities in US and Europe could also be influenced by the local culture to use E-B medicine. Reassuringly, CAM was typically used to ‘complement’ rather than ‘substitute’ for E-B medicine. Most studies recruited participants from healthcare centres effectively excluding carers/patients who avoid E-B medicine.^10,28^ In contrast, our study which recruited from primary schools in Malaysia—a country in which CAM-use is embedded within majority communities—identified a small, but important minority (4 of the 46 participants were classified as ‘CAM-users’) in whom CAM-use was preferred with E-B medicine being considered as a last resort.

A few accounts suggested that alternative ‘CAM-use’ might result in harmful practices either because of (potentially fatal) delay in use of E-B medicine during asthma attacks^31^ or dangerous CAM practices such as inducing vomiting. This practice would not only delay asthma treatment but also expose the child to the dangers of aspiration.^32^

A strength of our study is the broad range of opinions about E-B medicine and CAM encompassing committed CAM users, non-CAM users as well as those balancing the two approaches. We were concerned by the poor asthma control in this study population. However, there was no, or minimal, barrier to asthma care among the children as the Malaysian government provides free treatment for school children and the participants lived in areas that were accessible to government healthcare centres. Thus, the carers’ choice of medications was largely influenced by the carers’ view and perception of CAM and E-B medicine.

Our maximum variation sample of 46 participants enabled us to reach saturation with regard to beliefs about the two treatment modalities across the three cultural groups in Malaysia. We used a recognised framework devised to provide insight into the use of E-B medicine,^8^ and also applied it to perceptions of CAM enabling us to explore the relationship between the use of CAM and self-reported adherence to E-B medicine. There is a possibility that some of the children did not have asthma as we used parent-reported physician-diagnosed asthma to identify the children and we undertook no verification to confirm the diagnosis, however our questions about how parents used CAM and/or E-B medicine to manage their child’s symptoms remain valid.

The Tamil and Mandarin transcripts were translated and analysed in English, and there is a possibility that some meaning could be lost in translation, as the source language may not be fully represented in English. However, bilingual moderators checked each transcript to look for any loss of meaning during translation as well as supplying contextual meaning to minimise error. We were aware of reflexivity and the influence this might have on our data generation and analysis, but we undertook duplicate coding and discussed the findings in a multidisciplinary group to aid balanced interpretation.

With expanding cultural diversity around the world, healthcare professionals need to be aware of the different types of CAM-use and closely monitor the impact this may have on adherence to E-B medicine among their patients.

CAM use is engrained in society, and prohibiting its use is inappropriate and likely to be counter-productive if carers feel unable to disclose their use or even worse, are discouraged from seeing health professionals.^12,33,34^

Our study has important lessons for self-management support. Healthcare professionals should ask about the use of CAM, discuss beliefs about the necessity for and concerns about use of both E-B medicine and CAM, and provide balanced information about effectiveness and safety of E-B medicines. The aim is to improve adherence to regular E-B preventer medication and advise on safe practice in the event of deterioration.

Further research exploring the use of CAM and the influence on adherence to E-B medicine is needed in order to inform the development of interventions to improve the currently poor asthma control. Studies should focus on low and middle-income countries where the majority of the population use CAM and specifically include people who avoid E-B medicine.

In conclusion, this study has identified different profiles of CAM use, explored the reasons for CAM use, and shed light on the relationship of CAM use with adherence to E-B medicine. Greater CAM use and poor adherence with E-B medicine are associated with concerns about the safety of E-B medicine. Crucially, however, the key message which should be reiterated frequently and consistently is that CAM should be used to complement and not substitute for E-B medicine.^34,35^ The aim is to improve adherence to E-B preventer medication and prevent delays in seeking medical advice and harmful practices associated with CAM.

## Methods

### Regulatory approvals

This qualitative study was conducted at seven primary schools in Port Dickson, a suburban district in Malaysia with ethics approval from the Medical Research Ethics Committee in Malaysia (MECID: 20155–1366). The study is registered with the Malaysian National Medical Research Register (NMRR-15–1242–26898) and approval to conduct this study was obtained from the Ministry of Education, Malaysia.

### School recruitment

The schools were purposely chosen to represent the diversity of cultures. There are three main types of national primary schools in Malaysia; most use Malay as the first language, while others primarily use Mandarin or Tamil.^36^ Carers can choose their child’s school and often prefer to select according to language and culture.

### Identifying and recruiting children with asthma

We used a screening questionnaire to all the pupils in each school to identify children previously diagnosed with asthma. The questionnaire was taken home for completion by their carers. Maximum variation sampling was used to select carers with children from different age groups and ethnicities. We contacted selected carers, briefed them about the study and invited them to participate in a focus group discussion with an option of individual interviews for those unable or preferring not to attend focus groups. Participants provided written informed consent and were reimbursed their travel expenses. Concurrent focus groups and interviews were undertaken with the children, but we did not explore CAM with the children as we assumed that carers would be taking decisions about medication among primary school children; we are therefore not reporting details of the children’s aspect of the project.

### Data collection

Carers’ focus groups (*n* = 12 with up to seven participants and duration up to 68 min) and one individual interview were conducted (20 min). Basic socio-demographic data were collected from the participants using a questionnaire. We used a piloted semi-structured topic guide informed by previous literature and bio-psychosocial model^37^ to guide the focus groups and interviews (see Supplementary Note 1). The topic guide explored the experience, self-management and health beliefs of asthma specifically including the use of CAM and self-reported adherence to E-B medicine.

With the permission of the school, we timed our approach to the schools to minimise inference with school activities and ensure safety of the school children. We started the interviews in the schools using the Malay language, followed by Chinese (Mandarin) schools and Indian (Tamil) schools and the interviews were conducted in schools or primary care clinics according to participants’ convenience.

The interviews were conducted in the participants’ preferred language (Malay, Mandarin or Tamil), and moderated by researchers who were native speakers of each respective language. We used pictures of E-B medicines e.g., inhalers, aerochambers and nebuliser administration to facilitate recognition of medication or device used. All interviews were audio-recorded, and a note taker was present in each focus groups and interview to assist in transcribing and noting non-verbal expressions. There were intervals between interviews at different types of schools to allow basic analysis of the transcripts to be performed. This enables iterative modification to the topic guide for subsequent focus groups and interview. Analyses were performed iteratively until no new themes emerged.

### Data analysis

The focus groups and interview were transcribed verbatim and checked for accuracy. Experienced bilingual translators who were native speakers of the source language translated the Mandarin and Tamil transcripts into English. The bilingual interview moderators checked the translated transcripts for accuracy in translation and contextual meaning. The Mandarin and Tamil transcripts were analysed in English. The Malay transcripts were analysed in the source language as the researchers were fluent in Malay. Malay quotations used in this publication were translated to English using forward and backward translation process.

Three researchers (SNR, HS and NS) who were Malay native speakers coded the first Malay transcript independently and discussed the initial codings. The team discussed and agreed upon a coding framework, and SNR paired with a member of the team to code each transcript based on the coding framework. Emerging new codes were discussed with the team, and added to the coding framework. SNR compared all the coded transcripts for accuracy in coding and analysed using a thematic approach assisted by Nvivo software version 11. We used the Necessity-Concern Framework to inform the understanding of carer’s perception of and attitude to using E-B medicine and CAM.^38^ The quotes that best captured the essence of the themes were extracted for presentation in the results.

### Reflexivity and interpretation

SNR is a Malay primary care physician, a mother who has a child with asthma, has lived in Malaysia for more than 35 years and used CAM as supplements to E-B medicine. She does not discourage her patients from using CAM, encourages them to inform healthcare professionals about their use, and advises against the use of CAM if there is evidence that it may be harmful. HS is a primary care physician, and NS is a master student in health research. To ensure balanced interpretation, the results were discussed with the multicultural team which consisted of primary care physicians, a medical researcher and a paediatrician.

## Supplementary information

Supplementary Note 1

## Data Availability

The data that support the findings of this study are available from the corresponding author upon reasonable request.

## Supplementary information

**Supplementary information** accompanies the paper on the *npj Primary Care Respiratory Medicine* website (10.1038/s41533-019-0118-x).
