# Supplementary material for: Perceptions of complementary/alternative medicine use and influence on evidence-based asthma medicine adherence in Malaysian children
Source: NPJ Prim Care Respir Med. 2019 Feb 25;29:5. doi: 10.1038/s41533-019-0118-x (PMC6389881; doi:10.1038/s41533-019-0118-x)
Supplement: Supplementary file 1 — Supplementary Note 1 [file 41533_2019_118_MOESM1_ESM.pdf]

Childhood Asthma
Participant Interview Topic Guide (parents)

Preamble:

- **Ice-breaking and explain that there is no right or wrong answer**
- **Explain need to get consent for the interview and audio-recording. Obtain consents.**
- **Explain that the participant does not have to answer if he or she does not wish to do so**

Question	Prompts
**Asthma experience**	
What do you know about asthma?	Experience? Reading? Told by someone?
Tell us about your child's asthma? How is it now?	Cough, nightime cough, difficulty breathing?
How do you know if your child's asthma is good or bad?	Able to play? Easy breathing? No cough? **Where do you know about this?**
How does your child's asthma disturb/not disturb he/her? How? How does your child's asthma disturbs/not disturb you? How?	Activities affected e.g school? swimming, PJK, sleeping in fan? air conditioned room? Early morning or night bath? Play in the rain?
How does having asthma makes your child feel?	Friends isolation/bully- because of illness, using inhalers, shy? Okay?
How does your child's asthma makes you feel?	Anxious? Overprotective?
**Asthma self-management**	
What do you do when your child has asthma?	Inhaler? Go to hospital/clinic? Asthma action plan? **How do you know this?**
How about other treatment? Complementary or alternative medicine?	How do you use it? Why do you use it?
Who take care of your child's asthma at home? School?	You? Your child? Maid? What do you/they do?
Does your child take anything else other than these for his/her asthma? (showing inhalers and syrup)	Other medication from doctors?
**Asthma treatment**	
Tell us about your child's medicine.	Pills? MDI? Spacer? Aerochamber? Have you seen 'these' (peak flow meter) before? How do you use it? **When do you use your medication?How often?** Which medication is better? Reliever ? controller? How would you know when do your child needs the pump?

How do you feel about your child taking medicines?<br><br>How often do your child see the doctor for asthma?<br><br>When your child goes to clinic what was usually done?<br><br>What do you think about the clinic visits for asthma (clinic, doctor & medications)?<br><br>How do you feel when you accompany your child to clinic?	Dislike? Troublesome? Ashamed? **Why?**<br><br>Follow up? Emergency (hospital)? Stay in hospital ? When? Why?<br><br>Explanation? Medication?<br><br>Difficult access? Language barrier? Waiting time? Unfriendly staff?<br><br>Unpleasant? Okay?
**Health belief about asthma**<br><br>Why do you think your child has asthma?<br><br>**What triggers your child's asthma?**<br><br>What other things you do/eat for your child's asthma?<br><br>Do you have rules about asthma? (Things to avoid to prevent asthma, In Malay " Pantang")	 <br>Inherited? Infection? Environment?<br><br>**Dust? Physical activity? Cold weather?**<br><br>Home nebulizer? How does it help? Homeopathy? Which method is better for you? Why? **Where did you know this from? What does your child usually eats?**<br><br>Food, drinks or activities? Any other rules? Who makes the rules? **Experience? Or from somewhere? What will happen when they break the rules?**
**Source of information**<br><br>Where and from whom did you get information about asthma?<br><br>Anything else that you want to share about your child's asthma?	 <br>Clinic? Family? Friends? Internet? How good is the information?
